# Supplementary material for: Predicting mortality in critically ill patients with hypertension using machine learning and deep learning models
Source: Front Cardiovasc Med. 2025 Aug 8;12:1568907. doi: 10.3389/fcvm.2025.1568907 (PMC12370655; doi:10.3389/fcvm.2025.1568907)
Supplement: Supplementary file 1 [file Datasheet1.pdf]

Supplemental Table 1. Bivariate association between outcome and predictors.

Variable	odd ratio	p-value	95% CI
Sex			
Male (1)	0.972	0.224	[0.929, 1.018]
Female (0)	ref	ref	ref
Race			
African American	ref	ref	ref
White	0.745	< 0.001	[0.688, 0.806]
Hispanic	0.552	< 0.001	[0.478, 0.639]
Other	0.663	< 0.001	[0.578, 0.693]
Age, years	1.029	< 0.001	[1.027, 1.031]
Minimum creatinine	1.259	< 0.001	[1.236, 1.282]
Maximum creatinine	1.201	< 0.001	[1.182, 1.219]
Maximum heart rate	1.011	< 0.001	[1.010, 1.013]
Mean heart rate	1.007	< 0.001	[1.006, 1.009]
Minimum systolic bp	0.983	< 0.001	[0.982, 0.985]
Mean systolic bp	0.990	< 0.001	[0.989, 0.992]
Minimum diastolic bp	0.968	< 0.001	[0.966, 0.970]
Mean diastolic bp	0.980	< 0.001	[0.978, 0.982]
Minimum SpO2	0.996	0.222	[0.989, 1.003]
Mean SpO2	0.993	0.263	[0.980, 1.006]
Minimum hemoglobin	0.894	< 0.001	[0.885, 0.903]
Minimum prothrombin	1.062	< 0.001	[1.057, 1.066]
Maximum prothrombin	1.030	< 0.001	[1.027, 1.033]
Minimum respiratory rate	1.036	< 0.001	[1.031, 1.042]
Maximum respiratory rate	1.016	< 0.001	[1.013, 1.019]
Mean respiratory rate	1.062	< 0.001	[1.056, 1.069]
Maximum glucose	1.001		[1.001, 1.001]
Minimum platelet count	1.000	0.382	[1.000, 1.000]
Minimum calcium	0.959	0.003	[0.932, 0.986]
Minimum bicarbonate	1.009	0.001	[1.004, 1.015]
Maximum potassium	1.122	< 0.001	[1.089, 1.156]
Maximum blood urea nitrogen	1.025	< 0.001	[1.023, 1.026]
Maximum red blood cell count	0.594	< 0.001	[0.574, 0.615]
Minimum red blood cell count	0.730	< 0.001	[0.708, 0.753]
Mean red blood cell count	0.640	< 0.001	[0.619, 0.662]
Maximum white blood cell count	1.003	0.002	[1.001, 1.006]
Maximum sodium	1.000	0.964	[0.996, 1.004]
Minimum sodium	0.995	< 0.001	[0.994, 0.995]
Mean sodium	0.988	< 0.001	[0.985, 0.990]
Length of ICU stay (days)	1.136	< 0.001	[1.128, 1.144]
SOFA score	1.037	< 0.001	[1.035, 1.038]
APS-III score	1.034	< 0.001	[1.030, 1.038]

Supplemental Table 2. Multivariable logistic regression of backward selection.

Variables	Adjusted Odds ratio	P-value	95% confidence interval
Sex			
Male (1)	0.0586	0.028	[0.006, 0.111]
Female (0)	ref	ref	ref
Race			
African American	ref	ref	ref
White	-0.3904	< 0.001	[-0.482, -0.299]
Hispanic	-0.4724	< 0.001	[-0.634, -0.311]
Other	-0.4935	< 0.001	[-0.597, -0.390]
Ag, years	1.5370	< 0.001	[1.494, 1.582]
Minimum creatinine	1.1579	0.006	[1.043, 1.286]
Maximum creatinine	0.7360	< 0.001	[0.662, 0.818]
Mean heart rate	1.0414	0.005	[1.012, 1.072]
Minimum systolic bp	0.8688	< 0.001	[0.836, 0.904]
Mean systolic bp	1.0605	0.004	[1.019, 1.104]
Minimum hemoglobin	0.8545	< 0.001	[0.807, 0.905]
Minimum prothrombin	1.1912	< 0.001	[1.156, 1.227]
Minimum respiratory rate	1.0771	< 0.001	[1.043, 1.113]
Maximum respiratory rate	0.9295	< 0.001	[0.898, 0.962]
Mean respiratory rate	1.0900	< 0.001	[1.046, 1.135]
Maximum glucose	1.0479	0.001	[1.019, 1.077]
Minimum calcium	1.0327	0.027	[1.004, 1.063]
Minimum bicarbonate	1.1297	< 0.001	[1.098, 1.162]
Maximum blood urea nitrogen	1.2297	< 0.001	[1.184, 1.277]
Maximum red blood cell count	0.5673	< 0.001	[0.534, 0.601]
Minimum red blood cell count	1.7058	< 0.001	[1.571, 1.852]
SOFA Score	1.0711	< 0.001	[1.032, 1.112]
APS-III Score	1.6294	< 0.001	[1.565, 1.696]
Length of ICU stay	1.1565	< 0.001	[1.126, 1.188]

Supplemental Table 3. Multivariable logistic regression AUC

**Feature selection**	**AUC**										
	mean	Run 1	Run 2	Run 3	Run 4	Run 5	Run 6	Run 7	Run 8	Run 9	Run 10
Initial selection	0.7503	0.7529	0.7540	0.7489	0.7503	0.7506	0.7502	0.7473	0.7495	0.7513	0.7505
Backward selection	0.7498	0.7473	0.7525	0.7498	0.7498	0.7503	0.7411	0.7468	0.7469	0.7499	0.7578
